# Amino Acids of Seminal Plasma Associated With Freezability of Bull Sperm

**DOI:** 10.3389/fcell.2019.00347

**Published:** 2020-01-14

**Authors:** Muhammet Rasit Ugur, Thu Dinh, Mustafa Hitit, Abdullah Kaya, Einko Topper, Bradley Didion, Erdogan Memili

**Affiliations:** ^1^Department of Animal and Dairy Sciences, Mississippi State University, Starkville, MS, United States; ^2^Department of Animal Genetics, Kastamonu University, Kastamonu, Turkey; ^3^Department of Reproduction and Artificial Insemination, Selçuk University, Konya, Turkey; ^4^Alta Genetics, Inc., Watertown, WI, United States

**Keywords:** amino acids, seminal plasma, freezability, bull sperm, metabolomics

## Abstract

Sperm cryopreservation is an important technique for fertility management, but post-thaw viability of sperm differs among breeding bulls. With metabolites being the end products of various metabolic pathways, the contributions of seminal plasma metabolites to sperm cryopreservation are still unknown. These gaps in the knowledge base are concerning because they prevent advances in the fundamental science of cryobiology and improvement of bull fertility. The objective of this study was to test the hypothesis that seminal plasma amino acids are associated with freezability of bull sperm. To accomplish this objective, amino acid concentrations in seminal plasma from seven bulls of good freezability (GF) and six bulls of poor freezability (PF) were quantified using gas chromatography–mass spectrometry (GC–MS). Multivariate and univariate analyses were performed to identify potential freezability biomarkers. Pathways and networks analyses of identified amino acids were performed using bioinformatic tools. By analyzing and interpreting the results we demonstrated that glutamic acid was the most abundant amino acid in bull seminal plasma with average concentration of 3,366 ± 547.3 nM, which accounts for about 53% of total amino acids. The other most predominant amino acids were alanine, glycine, and aspartic acid with the mean concentrations of 1,053 ± 187.9, 429.8 ± 57.94, and 427 ± 101.3 nM. Pearson’s correlation analysis suggested that phenylalanine concentration was significantly associated with post-thaw viability (*r* = 0.57, *P*-value = 0.043). Significant correlations were also found among other amino acids. In addition, partial least squares-discriminant analysis (PLS-DA) bi-plot indicated a distinct separation between GF and PF groups. Phenylalanine had the highest VIP score and was more abundant in the GF groups than in the PF groups. Moreover, pathway and network analysis indicated that phenylalanine contributes to oxidoreductase and antioxidant reactions. Although univariate analysis did not yield significant differences in amino acid concentration between the two groups, these findings are significant that they indicate the potentially important roles of amino acids in seminal plasma, thereby building a foundation for the fundamental science of cryobiology and reproductive biotechnology.

## Introduction

There is an urgent need for more efficient, sustainable, and profitable cattle farming to feed the ever-increasing world population. Artificial insemination (AI) using cryopreserved sperm is a significant tool for the agri-food industry to improve modern animal production. The first attempt on sperm cryopreservation was made in 1776 (Royere et al., 1996); since then significant progress has been made using various cryoprotective agents and protocols during the last two centuries. Such progress, however, has not yet achieved the desired level of success because post-thaw survivability of sperm cells is disappointingly low, <50%, despite the best effort put forward in developing preservation techniques (Nijs et al., 2009). During cryopreservation, sperm cells undergo cellular and molecular changes, among which are membrane damage, oxidative stress, DNA fragmentation, reduced mRNA–protein interactions, as well as epigenetic modifications (O’Connell et al., 2002; Flores et al., 2011; Valcarce et al., 2013). Such modifications have detrimental effects on sperm physiology and thus on fertility.

Bovine seminal plasma is composed of secretions from testis, epididymis, and accessory sex glands. Such mixture contains proteins, ions, and metabolites including amino acids, lipids, monosaccharides, nucleosides, minerals, electrolytes, and steroid hormones (Egea et al., 2014; Cheng et al., 2015). As metabolites are the end-products of metabolic pathways, they play significant roles in sperm physiology such as energy metabolism, motility, and regulation of metabolic activities (Bieniek et al., 2016). While some components of seminal plasma have positive influences on sperm cryotolerance, others have detrimental effects (Yeste, 2016; Recuero et al., 2019). Regardless, metabolites in seminal plasma can be used to estimate bull fertility and sperm freezability. Hamamah et al. (1993) analyzed seminal plasma from fertile and infertile men using ^1^H nuclear magnetic resonance (NMR) spectra, and found significant differences in concentrations of glycerylphosphorylcholine citrate (GPC), and lactate between azoospermic and oligoasthenozoospermic patients. Lin et al. (2009) characterized the metabolite profiles of primate sperm to investigate the association between metabolism and energy supply. The association between glycolytic substrates and energy production, which is essential for motility, was determined using metabolomics approach in mouse spermatozoa (Goodson et al., 2012). More recently, using both NMR and gas chromatography–mass spectrometry (GC–MS), total of 96 metabolites and more than 10 biological pathways were identified in human sperm (Paiva et al., 2015).

Free amino acids of seminal plasma have various functions including reducing free radicals, protecting cells against denaturation, and providing oxidizable substrate to spermatozoa (Mann and Lutwak-Mann, 1981). However, identities and roles of seminal plasma amino acids during cryopreservation are not fully understood. Alanine, glycine, glutamine, histidine, and proline have been used as cryoprotectant agents for various species as they either inhibit lipid peroxidation or modulate osmotic mechanism (Heber et al., 1971; Renard et al., 1996; Trimeche et al., 1999; Jaiswal and Eisenbach, 2002; Dvořáková et al., 2005; Sangeeta et al., 2015). In addition to stabilizing proteins, amino acids also possess antioxidant properties to protect sperm cells from cold shock (Atessahin et al., 2008). For example, proline improves motility and protects sperm cells against damages caused by free radicals by stabilizing the membrane structure and function during the freezing (Rudolph et al., 1986; Smirnoff and Cumbes, 1989). Additionally, alanine and glutamine also affect the motility and viability of the sperm (Koskinen et al., 1989; Khlifaoui et al., 2005; Amirat-Briand et al., 2009) by to some extent improving the cryoprotective effects of glycerol.

Recently, we have identified 63 seminal plasma metabolites of which 21 were amino acids from bulls with different field fertility scores (Velho et al., 2018) demonstrating the importance of metabolite profiles between low and high fertility bulls. Seminal plasma addition before freezing also influenced on post-thaw bull sperm kinematics (Nongbua et al., 2018). To investigate further the impacts of seminal plasma composition on sperm cells, in this study we ascertained the relationship between freezability and amino acids in bull seminal plasma.

## Materials and Methods

### Semen Collection and Determination of Bull Semen Freezability

Seminal plasma samples from 13 bulls with various freezability and semen freezability data were provided by a commercial breeding company (Alta Genetics Inc., Watertown, WI, United States). The bulls were housed in the same nutrition and management environment to prevent sample variation. Semen was collected using artificial vagina and protease inhibitor was added immediately. Semen was then centrifuged at 800 × *g* for 15 min to separate the seminal plasma and sperm. This seminal plasma was transferred into sterile microcentrifuge tubes and centrifuged again at 800 × *g* for 15 min to completely eliminate sperm in the sample. Following this second centrifugation, seminal plasma was transferred into new tubes and shipped to Mississippi State University (MSU) in a liquid nitrogen tank.

Bull semen was extended with commercial egg-yolk–tris-based extender, and then frozen at Alta Genetics using standard protocols (Pace et al., 1981). Briefly, fresh semen was collected from bulls via artificial vagina, and semen was evaluated for concentration, volume, color, and motility. Then, semen was extended with one-step egg-yolk–tris–glycerol extender. The extender included 20% egg-yolk and 6% glycerol. This is called initial extension which includes fourfold dilution with extender at 32°C. The extended semen was cooled down to 5°C within 90 min. Then, the remaining extender was added at 5°C to complete extension, and packaged into quarter cc straws (250 μl) and let semen equilibrate for 3–4 h. Following the equilibration process, straws were frozen using automated freezer machine. The freezing was completed within 14 min (temperature from 5°C to −196°C), and stored in a liquid nitrogen tank.

Post-thaw sperm viability was assessed using flow cytometry (CyFlow SL, Partec, Germany). Fluorescent stain combinations of SYBR-14 with propidium iodide (SYBR-14/PI, Live/Dead Sperm Viability Kit L-7011, Thermo Fisher Scientific) were used as described previously (Garner et al., 1994; Nagy et al., 2004). Membrane integrity of 10,000 sperm cells from each collection was measured with the highest accuracy and objectivity. We verified that biological sample preparations, instrument configurations, and data analysis were compliant with the recommendations set by the International Society for Advancement of Cytometry on the minimum information necessary. The CyFlow SL (Partec, Münster, Germany) instrument equipped with 488 nm blue state laser allowed excitation of SYBR14 and PI to measure sperm viability. It was also fully equipped with five parameters: FSC, SSC, red, green, and orange/yellow colors. With the Partec FloMax software, the instrument allowed a real-time data acquisition, data display, and data evaluation.

The quality control measures and repeatability of flow cytometric sperm viability analysis were routinely verified by control samples which consisted of positive (100% dead sperm) and negative control (100% live sperm) of standard biological samples and their mixture of different ratios (100/100, 75/25/50/50, 25/75, and 100/100% dead and live sperm combinations). Another quality measure we used was the control of reagents (SYBR-14 and PI). The reagents and biological standards were used to calibrate the instrument settings and data processing. In the calibration, non-sperm particles were gated out and not included in the calculations. Partially stained (green and red) moribund sperm were considered as dead in the analysis. The percentage of live (green) sperm is used as a measure of sperm freezability parameter, the percentage of sperm that maintained membrane integrity during freeze–thawing process. The following formula was used to count the percentage of viable sperm: The% Viable sperm = The number of viable sperm/Total sperm (viable + dead + moribund) × 100.

Collectively, a unique freezability phenotype was generated to characterize variation among bulls for their lifetime post-thaw viability of sperm. For this particular research, we used post-thaw viability data generated over 8 years period (between 2008 and 2016). The database included 100,448 ejaculates from 860 Holstein bulls that were collected at least 20 different times in approximately 3 months period. The average and standard deviation of post-thaw viability for individual bulls were calculated, and then bulls were ranked based on average post-thaw semen viability. The threshold was the population average which consisted of the 100,448 ejaculates from 860 Holstein bulls. The average post-thaw viabilities of all bulls ranged from 33.03 to 67.3% (population average 54.7 ± 5.4%). The bulls were then classified as GF and PF based on average post-thaw viability score and the differences from the population average. The population average was our threshold to classify GF and PF groups. Bulls with greater sperm post-thaw viability than population average grouped as GF while those lower than average were considered as PF. Total of 13 bulls were selected with high confidence among 860 bulls for the presented study (Table 1).

**TABLE 1:** Semen freezability phenotypes of the Holstein bulls used for GC–MS analysis: **(A)** Bulls 1–7 were defined as good freezability (GF) and Bulls 8–13 were grouped as poor freezability (PF) and **(B)** Percent differences of good and poor freezing phenotypes from the population average.

**(A)**			
**Bull No.**	**Freezability status**	**Average post-thaw viability (%)**	**Difference from population average (%)**

1	Good freezability	66.19	11.50
2		64.40	9.71
3		64.28	9.59
4		62.34	7.65
5		61.95	7.26
6		59.92	5.23
7		58.37	3.68
8	Poor freezability	55.03	0.34
9		54.92	0.23
10		54.77	–0.08
11		52.68	–2.01
12		49.23	–5.46
13		48.93	–5.76

**(B)**			

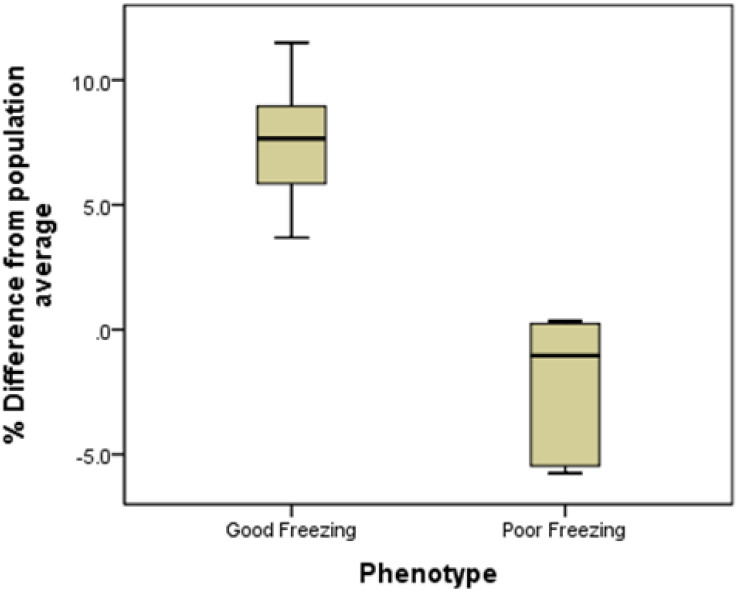			

*Bulls were classified as good freezability and poor freezability based on average post-thaw viability scores and the percent differences from the population average (*P* < 0.001).*

### Sample Preparation for Gas Chromatography–Mass Spectrometry Analysis

The amino acid analyses of seminal plasma from 13 bulls with various freezability were performed using EZfaast Amino Acid kit (©Phenomenex Inc., Torrance, CA, United States) as previously described by Kaspar et al. (2008). All samples were prepared and analyzed according to protocol that provided by Phenomenex Inc., Torrance, CA, United States. Briefly, 10 μl of seminal plasma and 25 μl of internal standard solution (norvaline 0.02 nM and *N*-propanol 10%) were pipetted into glass sample preparation vials. Solution in the sample preparation vial was passed through the sorbent tip using a syringe. Then, 200 μl of *N*-propanol was pipetted into the same vial and passed through the sorbent tip and into syringe barrel. Drained liquid from sorbent tip was discarded. One hundred and twenty microliters of sodium hydroxide and 80 μl of *N*-propanol were pipetted into same glass vial, and the particles inside the sorbent tip were ejected into solution. A volume of 50 μl of chloroform–propyl chloroformate, and 100 μl of iso-octane were transferred to the tube and the resulted mixture was vortexed for 1 min after each adding. Transparent part of the (upper) organic layer transferred into autosampler vial, and evaporation of the solvent was achieved using a TurboVap^®^ LV evaporator (Biotage, Charlotte, NC, United States) with a gentle stream of nitrogen at 30°C. The extract was then suspended in 50 μl of solution containing iso-octane (80%) and chloroform (20%) and transferred to an amber glass vial having a fixed insert (Agilent Technologies, Santa Clara, CA, United States) for the analysis using GC–MS.

### Amino Acid Analysis Using Gas Chromatography–Mass Spectrometry

We used recommended GC-MS parameters to analyze the seminal plasma amino acids and the reference standards in an Agilent 7890A GC System that was coupled to an Agilent 5975C inert XL MSD with triple-axis mass detector, an Agilent 7693 Series Autosampler, and a capillary column (Zebron^TM^ EZ-AAA 10 m × 0.25 mm; ©Phenomenex, Santa Clara, CA, United States). The derivatized mixture (1.5 μl) was injected into the inlet that was heated at 250°C with 1:15 split ratio. Following the injection of the sample at 3 ml/min, standard septum purge was performed using helium carrier gas at 1 ml/min constant flow rate. Auxiliary, ion source, and quadrupole were heated at 310, 240, and 180°C, respectively. The oven was programmed initially at 110°C, and ramped up to 320°C within 11 min. The solvent delay time was at 1.30 min. The MS was operated in selected ion monitoring (SIM) mode and appropriate ion sets were selected. All amino acids were identified based on retention times, target and qualifier ions in comparison with authentic standards supplied by ©Phenomenex Inc., Torrance, CA, United States (Table 2). For calibration, increasing volumes of the diluted standards (0, 5, 10, 40, 80, 160, and 200 nmol/ml) were as described above. Abundances of the target ions of amino acids were divided by abundance of target ion of the internal standard (norvaline) and the unitless ratios were used to calculate amino acid concentrations using internal standard calibration.

**TABLE 2:** Selected ions and retention times for the SIM analysis of 22 amino acids, dipeptides and internal standard (norvaline).

**Amino acid**	**Abbreviation**	**Retention time**	**Target ion (*m*/*z*)**	**Qualifier ion (*m*/*z*)**
Alanine	ALA	1.42	130	88
Glycine	GLY	1.53	116	207
Alpha-aminobutyric acid	ABA	1.64	144	102
Valine	VAL	1.74	158	116
Beta-aminobutyric acid	Beta-AiB	1.83	158	116
Norvaline	NOR	1.88	158	72
Leucine	LEU	1.97	172	76
Allo-isoleucine	aILE	2.00	172	130
Isoleucine	ILE	2.03	172	130
Threonine	THR	2.25	160	101
Serine	SER	2.29	146	203
Proline	PRO	2.37	156	243
Asparagine	ASN	2.47	155	69
Aspartic acid	ASP	3.04	216	130
Methionine	MET	3.08	203	277
4-Hydroxyproline	4HYP	3.22	172	86
Glutamic acid	GLU	3.42	230	170
Phenylalanine	PHE	3.45	206	190
Alpha-aminoadipic acid	AAA	3.73	244	98
Ornithine	ORN	4.48	156	70
Lysine	LYS	4.75	170	128
Tyrosine	TYR	5.24	206	107
Tryptophan	TRP	5.54	130	

*All amino acids were identified based on retention time, target and qualifier ions.*

### Statistical Analysis

The associations between freezability of sperm and concentration of seminal plasma amino acids were assessed using both univariate and multivariate approaches. For univariate approach, a generalized linear mixed model was used to determine the statistical significance between GF and PF groups. The variance was estimated by the GLIMMIX procedure of SAS 9.4 (SAS Institute Inc., Cary, NC, United States). The Kenward–Roger approximation was used to calculate the degree of freedom in case of heterogeneous variances. In addition, correlations among seminal plasma amino acids and correlations between seminal plasma amino acid concentrations and freezability scores were determined using Pearson’s correlation (Xia and Wishart, 2011). For multivariate analyses, MetaboAnalyst 3.0^1^ (Xia et al., 2015) was used. For each variable, an observation was subtracted from the overall mean and the difference was divided by the standard deviation. This scaling or normalization of the data allowed us to bring the variances of all variables to the value of 1 while preserving the relative variability among observations within a variable. Following the normalization of data, partial least squares regression-discriminant analysis (PLS-DA) was performed and the bi-plot was constructed. The VIP scores in PLS-DA were calculated to identify significance of variables on phenotype. A VIP score >1.5 was considered as significant for group separation, and the significance level of 0.05 was used to determine statistical significance for other analyses. GraphPad Prism 8 was used (GraphPad Software, Inc., La Jolla, CA, United States) to generate some of the figures.

### Pathway and Network Analysis

Pathways and network analyses of amino acids were performed using bioinformatic tools. The pathway-based compound-reaction-enzyme-gene networks were identified using MetScape 3.1 (Karnovsky et al., 2012) which was plug in Cytoscape 3.7.1^2^. Interactomes of gene products defined by MetScape 3.1 were identified using the biological networks gene ontology tool (BiNGO) within Cystoscope 3.7.1. A merged network was created in Cystoscope by entering subjected genes to analyze the interactome of genes for Bos Taurus, and significance level was set as 0.05.

## Results

### Amino Acid Concentration in Bull Seminal Plasma

**FIGURE 1:**
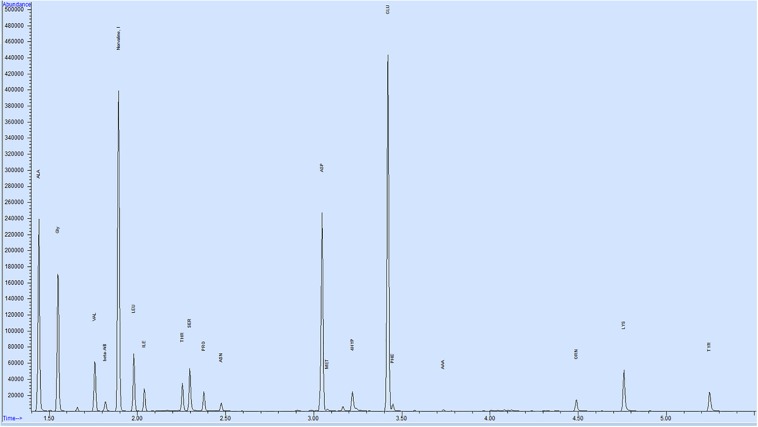
Representative GC–MS chromatogram of bull seminal plasma. Total of 21 amino acids were identified using SIM (selected ion monitoring), Norvaline used as an internal standard with a concentration of 200 nM.

**FIGURE 2:**
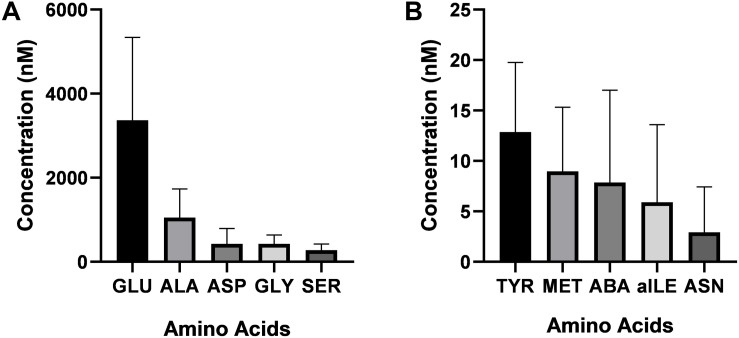
Concentrations of the most and the least abundant amino acids in bull seminal plasma. **(A)** The most abundant amino acids in bull seminal plasma was glutamic acid. Alanine, glycine, aspartic acid, and serine were the other predominant amino acids in bull seminal plasma. **(B)** The least predominant amino acids were tyrosine, methionine, alpha aminobutyric acid, allo-isoleucine, and asparagine.

Twenty-one amino acids were detected in bull seminal plasma (Figure 1). Free amino acid concentrations of bull seminal plasma are depicted in Table 3. Among these, glutamic acid was the most abundant amino acid in seminal plasma with an average concentration of 3366 ± 547.3 (mean ± SD) nM, which accounts for approximately 53% of all the amino acids. The other most predominant amino acids were alanine, glycine, aspartic acid, and serine with mean concentrations of 1053 ± 187.9, 429.8 ± 57.94, 427 ± 101.3, and 278.2 ± 40.14 nM, respectively (Figure 2A). The least abundant were tyrosine, methionine, alpha-aminobutyric acid, allo-isoleucine, and asparagine with mean concentrations of 12.87 ± 1.91, 8.97 ± 1.76, 7.87 ± 2.53, 5.91 ± 2.13, and 2.92 ± 1.25 nM, respectively (Figure 2B).

**TABLE 3:** Average amino acid concentrations (nM) of seminal plasma from good and poor freezability semen (mean ± SD).

**Amino acid**	**Good freezability**	**Poor freezability**	**Both**
Alanine	943.4 ± 232.9	1180.00 ± 318.6	1053 ± 187.9
Glycine	464.7 ± 90.62	389.10 ± 73.09	429.8 ± 57.94
α-Aminobutyric acid	6.349 ± 3.17	9.65 ± 4.258	7.874 ± 2.53
Valine	145.3 ± 13.89	134.80 ± 33.49	140.5 ± 16.43
β-Aminobutyric acid	92.77 ± 13.81	94.03 ± 25.50	93.35 ± 13.28
Leucine	140.7 ± 15.73	141.00 ± 26.44	140.8 ± 14.18
Allo-isoleucine	4.70 ± 1.67	7.32 ± 4.348	5.912 ± 2.13
Isoleucine	54.06 ± 5.93	45.66 ± 13.38	50.19 ± 6.73
Threonine	104.3 ± 19.67	149.10 ± 36.93	125 ± 20.20
Serine	250.2 ± 52.74	310.90 ± 63.93	278.2 ± 40.14
Proline	35.56 ± 9.34	46.87 ± 17.39	40.78 ± 9.18
Asparagine	1.98 ± 1.90	4.02 ± 1.63	2.92 ± 1.25
Aspartic acid	412.0 ± 120.3	444.40 ± 181.7	427 ± 101.3
Methionine	7.851 ± 1.99	10.27 ± 3.16	8.97 ± 1.76
4-Hydroxyproline	49.50 ± 9.74	44.45 ± 5.21	47.17 ± 5.59
Glutamic acid	3567 ± 899.8	3131.00 ± 635.6	3366 ± 547.3
Phenylalanine	25.53 ± 2.06	18.72 ± 1.28	22.38 ± 1.56
α-Aminoadipic acid	18.21 ± 3.23	16.23 ± 1.53	17.29 ± 1.83
Ornithine	33.39 ± 4.83	27.27 ± 3.98	30.56 ± 3.18
Lysine	77.43 ± 13.63	58.42 ± 12.76	68.65 ± 9.42
Tyrosine	14.25 ± 2.71	11.26 ± 2.78	12.87 ± 1.91

### Identification of Potential Freezability Biomarkers

**FIGURE 3:**
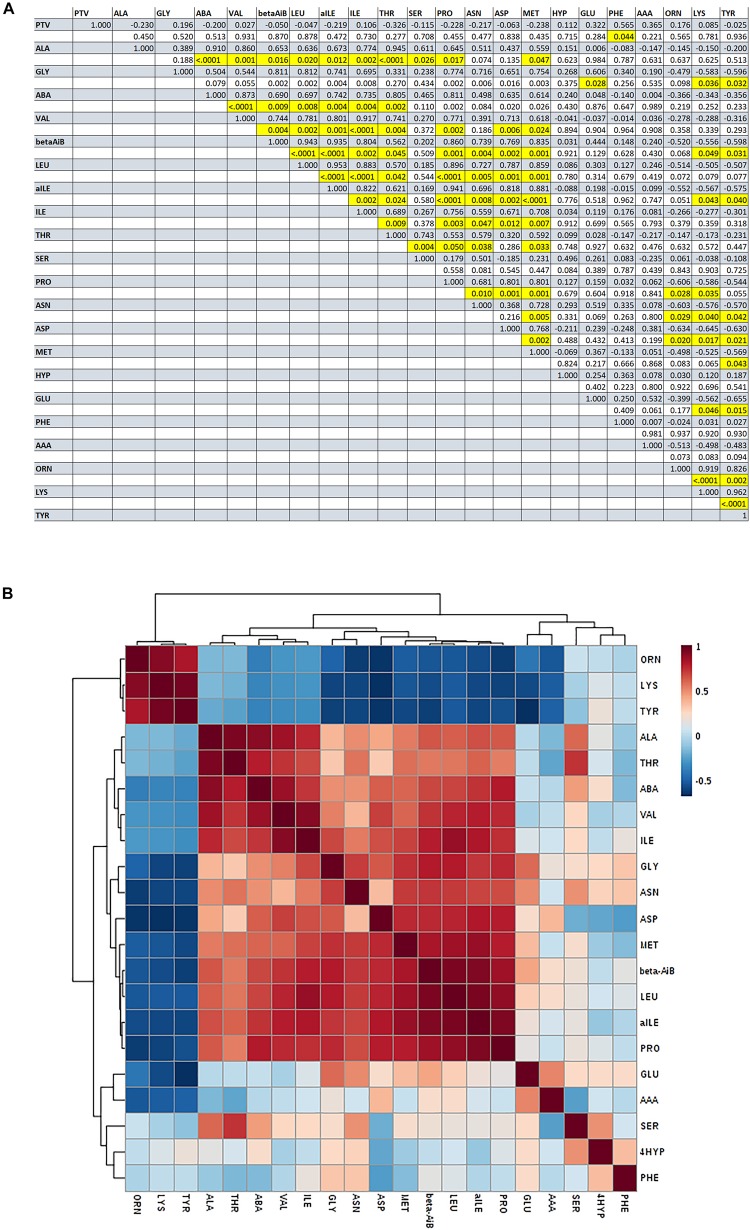
Pearson’s correlations among amino acids identified in bull seminal plasma. **(A)** Correlation matrix of amino acid concentrations in seminal plasma [shaded lines: Pearson correlation coefficients (*r*); white boxes: *P*-value; highlighted boxes: *P* < 0.05). **(B)** Heatmap of Pearson’s correlations among amino acids identified in bull seminal plasma.

There was no significant difference in amino acid concentrations between GF and PF groups (*P* > 0.05). However, phenylalanine concentration was significantly correlated with average post-thaw viability (*r* = 0.57, *P-*value = 0.044). Additionally, there were significant correlations among individual amino acids (Figure 3), such as the concentration of proline was positively correlated with leucine (*r* = 0.90, *P*-value < 0.0001), iso-leucine positively correlated with valine (*r* = 0.92, *P*-value < 0001), and the concentration of threonine was positively correlated with alanine (*r* = 0.95, *P-*value < 0001).

**FIGURE 4:**
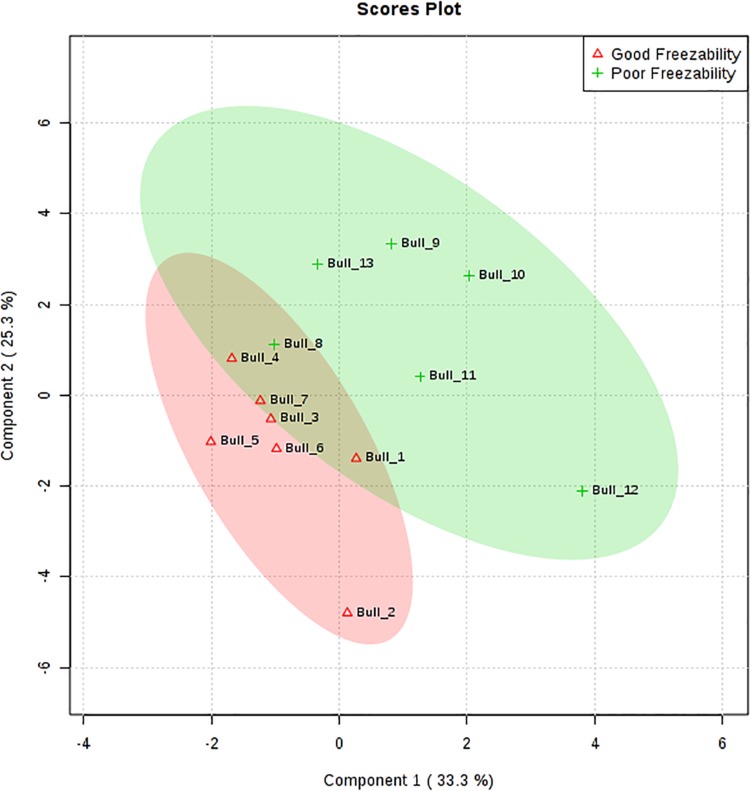
Partial least squares discriminant analysis (2D PLS-DA) of the seminal plasma amino acids from good freezability (GF) and poor freezability (PF) bulls. The plots indicate a separation between GF and PF bulls. PLS-DA was obtained with two components.

**FIGURE 5:**
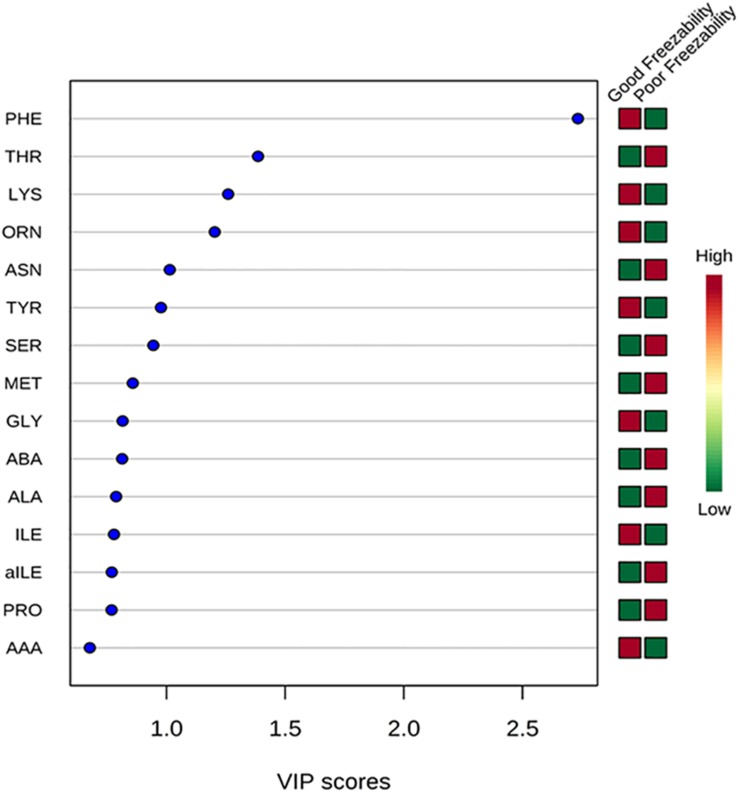
Variable importance in projection (VIP) plot displays the top 15 most important amino acid features identified by PLS-DA. Colored boxes on right indicate concentration of corresponding amino acid from GF and PF samples. VIP score is a weighted based on PLS-DA model.

The multivariate analysis, PLS-DA, of seminal plasma amino acids showed a distinct separation between GF and PF bulls (Figure 4). PLS-DA was used for the classification. A variable importance in projection (VIP) score, which is widely used in PLS-DA, rank the amino acids considering their significance in discrimination between the GF and PF bulls. VIP score is referred as a weighted sum of squares of the PLS loadings. The *X*-axis specifies the VIP scores to each variable on the *Y*-axis. Therefore, amino acids with VIP score >1.5 was identified as phenylalanine, and VIP score in the corresponding heat map demonstrated that phenylalanine is more abundant in seminal plasma of the GF bulls than in that of PF bulls (Figure 5).

### Pathways and Networks of Seminal Plasma Amino Acids

**FIGURE 6:**
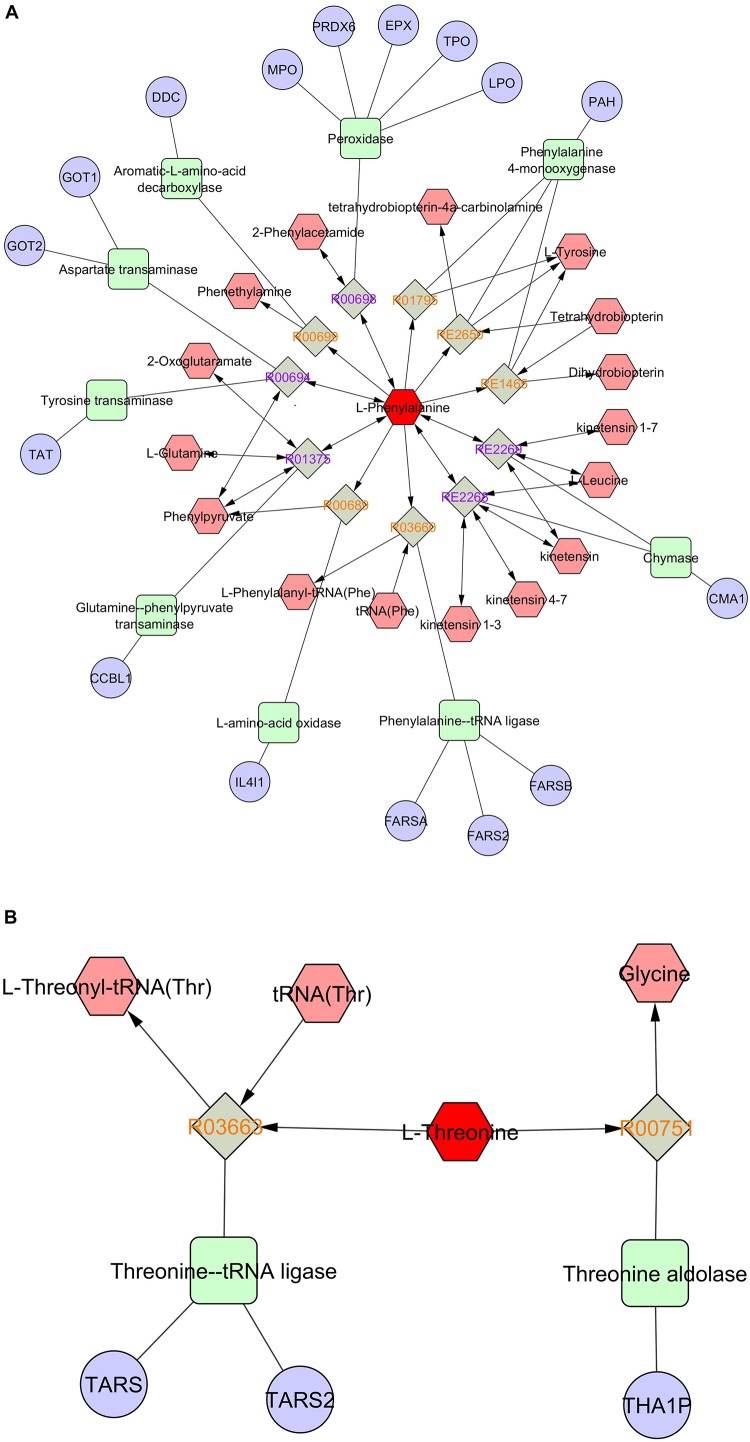
NOTHINGCOMESHERE

Pathway and network analyses of the amino acids with highest VIP scores (phenylalanine and threonine) and the most abundant amino acids (glutamic acid, alanine, and glycine) were performed using MetScape (3.1.3) (Karnovsky et al., 2012). By analyzing the results, we showed that phenylalanine was involved in tyrosine metabolism, and interacted with several compounds and genes (Figure 6A). The interactome of phenylalanine showed that this amino acid contributes to a number of cellular and biological processes, such as antioxidant detoxification, metabolic processes of reactive oxygen species, and oxidoreductase activity (Table 4). Threonine was involved in glycine, serine, alanine, and threonine metabolism and it shows significant gene ontology in terms of cellular amino acids and derivative metabolic processes (Figure 6B). Glutamic acid was correlated with many genes, enzymes, and other reactions (Figure 6C), most of which occur in mitochondria. It has a significant interactome regarding oxidoreductase activity, regulation of cell death, and the oxoacid metabolic process. It also contributes to histidine metabolism, the urea cycle, and the metabolism of arginine, proline, glutamate, aspartate, and asparagine, and Vitamin B9 (folate) metabolism. Alanine created a significant gene ontology in terms of ligase activity and forming carbon–oxygen bonds, and is also involved in pathways of glycine, serine, alanine, and threonine metabolism (Figure 6D). Finally, glycine is involved in seven different biological and cellular pathways, and has generated significant gene ontology in terms of oxidoreductase activity (acting on the CH-NH_2_ group of donors), sarcosine oxidase activity, and D-amino-acid oxidase activity. All findings are summarized in Table 4.

**FIGURE 7:**
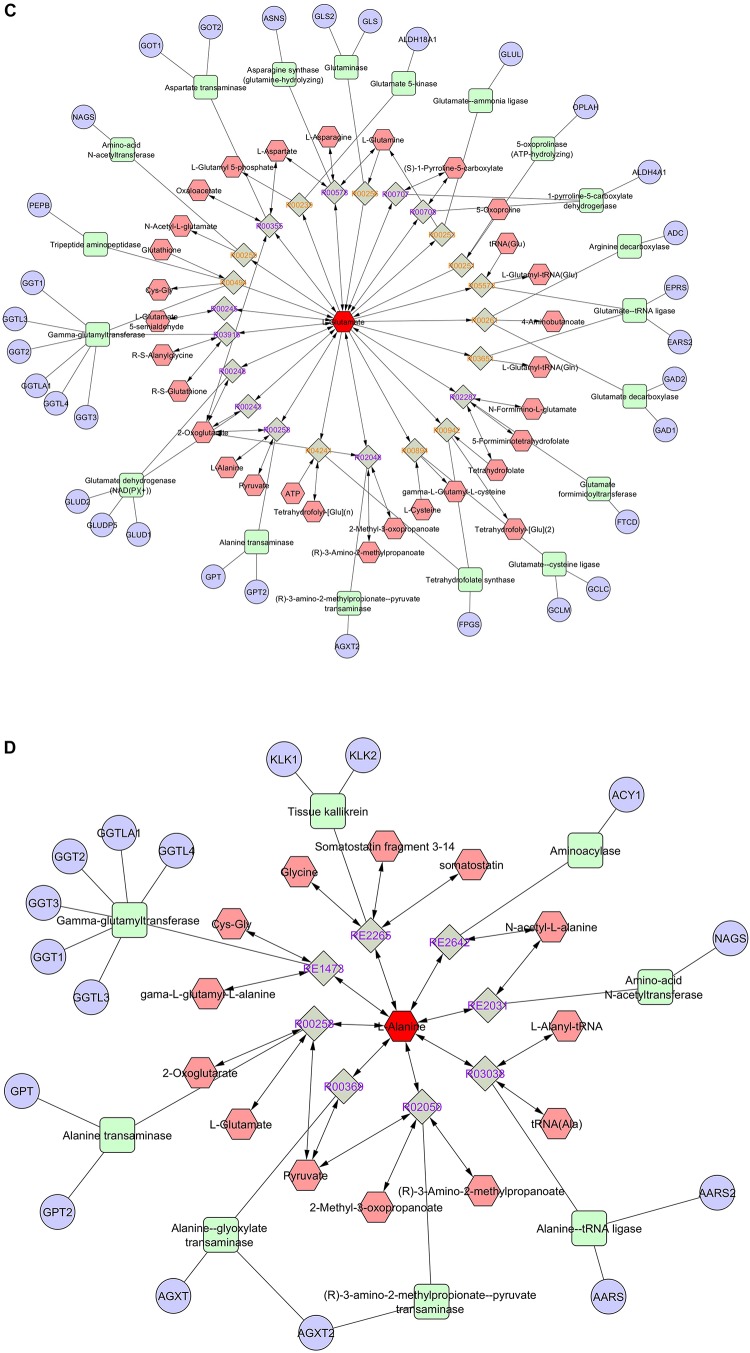
Pathway and network analyses of the amino acids with highest VIP scores (phenylalanine, threonine) and the most abundant amino acids (glutamic acid, alanine) were performed using MetScape. **(A)** Phenylalanine, **(B)** threonine, **(C)** glutamate, and **(D)** alanine. Amino acids are shown in red hexagons. Gray square: Reaction node with reaction ID; Pale red hexagon: Compound node; Green square: Enzyme node; Blue circle: Gene node.

**TABLE 4:** The interactome of amino acid shows that amino acid contributes to a great number of cellular and biological processes, such as antioxidant detoxification, reactive oxygen species metabolic processes, and oxidoreductase activity.

**Amino acid**	**GO ID**	**Description**	***P-*value**
Phenylalanine	10602	Oxidoreductase activity, acting on paired donors, with incorporation or reduction of molecular oxygen, reduced pteridine as one donor, and incorporation of one atom of oxygen	0.003
	10636	Cellular biosynthetic process	0.007
	10652	Oxygen and reactive oxygen species metabolic process	0.025
	10677	Response to stress	0.016
	10678	Response to oxidative stress	<0.0001
	10680	Tyrosine metabolic process	0.004
	10733	Oxidoreductase activity, acting on peroxide as acceptor	<0.0001
	10735	Response to reactive oxygen species	<0.0001
	10738	Antioxidant activity	<0.0001
	10788	Hydrogen peroxide metabolic process	0.012
	10789	Hydrogen peroxide catabolic process	0.007
	10865	Vitamin binding	<0.0001
	10879	Peroxiredoxin activity	0.007
	10894	Oxidation reduction	0.001
	10913	Cellular response to reactive oxygen species	0.013
	11069	Fatty acid transport	0.008
Threonine	11913	Cellular amino acid and derivative metabolic process	0.017
Glutamic acid	8483	Transaminase activity	<0.0001
	16639	Oxidoreductase activity, acting on the CH-NH_2_ group of donors, NAD or NADP as acceptor	0.001
	12782	Regulation of cell death	0.047
	43436	Oxoacid metabolic process	<0.0001
Alanine	15707	Ligase activity, forming carbon–oxygen bonds	0.017
	15722	Small molecule metabolic process	0.008
Glycine	16641	Oxidoreductase activity, acting on the CH-NH group of donors, oxygen as acceptor	0.001
	8115	Sarcosine oxidase activity	0.001
	3884	D-Amino-acid oxidase activity	0.003
	17834	Oxidoreductase activity, acting on the CH-NH_2_ group of donors	0.016

## Discussion

Successful sperm cryopreservation is an imperative element of fertility management and assisted reproductive studies (ART). The contributions that seminal plasma metabolites have on sperm cryopreservation are still largely unknown. In this present study, we performed GC–MS analyses to investigate the amino acid profiles of bull seminal plasma and classify potential biomolecular markers of freezability. Consecutively, bioinformatic tools were used to identify network and biological pathways of seminal plasma amino acids. To the extent of our knowledge, our study is the first to conduct an extensive assessment of amino acids in bull seminal plasma considering association of specific seminal plasma amino acids with freezability phenotypes.

Seminal plasma is a complex fluid composed of a broad range of metabolites such as organic compounds and energy substrates. Biochemical compositions of seminal plasma differ among species and even among individual males (Killian et al., 1993). This may be due to different management and feeding variations as well as metabolic activity of sperm. These metabolites in seminal plasma have functional roles in sperm preservation, motility, and control of metabolic activity (Bieniek et al., 2016). Amino acids and peptides are the major biochemical compounds found in bovine sperm and its seminal plasma. There is a wide range of amino acids in seminal plasma of which concentrations of many rise after ejaculation due to the massive proteolytic activities occurring in semen (Mann, 1964). Amino acids function as oxidizable substrates for the energy supply, causing reactions in semen (Neumark and Schındler, 2007).

The most abundant amino acid present in seminal plasma is glutamic acid accompanied by a considerable level of glutamic oxaloacetic transaminase (GOT) activity (Flipse, 1960). As in earlier bull semen studies, the abundance of glycine, alanine, serine, aspartic acid, and glutamic acid is found to be high and high levels of amino acids in seminal plasma are higher than in sperm (Roussel and Stallcup, 1967). In a recent study aimed at analyzing metabolomes of seminal plasma from bulls with somewhat higher vs. lower fertility, researchers have identified 63 metabolites, in seminal plasma, of which 21 are amino acids that can be potential biomarker of fertility. Abundances of L-leucine and ornithine differed between the fertility groups, and the levels of fructose were correlated with those of glutamic acid and amino-butyrolactone (Velho et al., 2018). In other studies, researchers have determined different numbers of amino acids and peptides, in seminal plasma of bull, where glutamic and aspartic acid were the most abundant, and were associated with fertility and pregnancy rates (al-Hakim et al., 1970; Holden et al., 2017). Also, seminal plasma of human and other species were found to contain large numbers of amino acids (Engel et al., 2019; Santiago-Moreno et al., 2019). Fertility and sperm freezability are not always related. This current study was aimed at ascertaining seminal plasma amino acids associated with sperm freezability. In the current study, we identified the glutamic acid as the most abundant amino acid. We also demonstrated that glutamic acid was correlated with a number of genes, enzymes, and other reactions, most of which occur in mitochondria. This provides an important evidence of interactome regarding oxidoreductase activity, regulation of cell death, the oxoacid metabolic process, and significant possibility of influence on cell energy production.

The other most predominant amino acids revealed in our study were alanine, glycine, aspartic acid, and serine. When these amino acids in seminal plasma were compared to those found in human seminal plasma (Li et al., 2019), profiles of some seminal plasma amino acids were similar to those profiles we found such as serine, glycine, and glutamic acids. The least abundant in our study, on the other hand, are tyrosine, methionine, alpha-aminobutyric acid, allo-isoleucine, and asparagine, and are found to be similar with the low levels of amino acids of methionine and tyrosine in bull (Assumpção et al., 2005). Also, in domestic fowl, valine, serine, glycine, and alanine were the most abundant amino acids followed by glutamic acid (Santiago-Moreno et al., 2019). The alanine created a significant gene ontology in terms of its ligase activity and formed carbon–oxygen bonds, and is also involved in pathways of glycine, serine, alanine, and threonine metabolism.

During the cryopreservation process, sperm undergo critical cryo-injury based on membrane damage, oxidative stress, and DNA fragmentation which reduce post-thaw viability of sperm cells. Even though the exact cryo-protectant mechanisms of amino acids have not been clearly understood, it is presumed they may bind phospholipid membrane bilayers and stabilize the cell membranes (Bilodeau et al., 2001). In addition, osmo-regulative and antioxidative features may provide resilience during freezing–thawing (Kruuv and Glofcheski, 1992; Farshad and Hosseini, 2013). However, there are not a great number of studies that have investigated the protective influence of amino acids against cryo-injury. Previous studies have claimed that seminal plasma supplementation of amino acids into semen extenders improved sperm viability, acrosome integrity and membrane integrity of sperm (Ali Al Ahmad et al., 2008), and post-thaw semen quality (Saravia et al., 2009). More specifically, in human research, it was found that addition of glutamine to semen as a cryoprotectant agent increased post-thaw motility in human sperm (Atessahin et al., 2008). In animal studies, supplementation of extender solutions with glutamine, glycine, and cysteine enhanced acrosome and membrane integrity of buffalo bull semen (El-Sheshtawy et al., 2008). Additionally, there was a positive correlation between membrane integrity and the concentration of valine, isoleucine and leucine, and lysine (Santiago-Moreno et al., 2019).

One of the most common negative consequences of cryopreservation of sperm cell is DNA damage, and majority of DNA lesions in sperm cells is caused by oxidative stress (Zribi et al., 2010). Seminal plasma content plays a significant role in protection against oxidative stress. Aitken and Baker (2004) clarified that taurine and hypotaurine are the amino acids that reduce oxidative stress through binding to the oxidizing agents. In addition, supplementation of donkey semen with glutamine reduced DNA fragmentation index (Bottrel et al., 2018). Sangeeta et al. (2015) reported that supplementation of ram sperm with L-glutamine and L-proline reduced lipid peroxidation and increased acrosomal integrity. Glutamic acid is the key component of glutathione which has been demonstrated to inhibit cellular damage resulting from lipid peroxidation and reactive oxygen species (Arai et al., 1999). In the present study, we showed that phenylalanine is more abundant in seminal plasma of the GF bulls than in that of PF bulls. It has significant gene ontology terms for antioxidant activity, response to oxidative stress, and oxidoreductase activity through its actions on peroxide as acceptor, and metabolic processes of oxygen as well as reactive oxygen species. We postulate that phenylalanine could have an antioxidant effect, and increased concentrations of phenylalanine in seminal plasma may reduce DNA damage caused by oxidative stress. Moreover, PLS-DA results demonstrate a distinct separation between GF and PF groups. Thus, the abundance of glutamic acid may explain protective effects of seminal plasma during cryopreservation. Furthermore, glutamine may play an important role in gene expression redox-potential, and cell integrity (Curi et al., 2005). It has been assumed electrostatic interactions between plasma membrane phospholipids and amino acids help to generate a layer on the sperm surface, and which thus protects the sperm cell from cryo-injury (Anchordoguy et al., 1988; Kundu et al., 2001).

## Conclusion

We have found that glutamic acid, alanine, and glycine are the predominant metabolites in bull seminal plasma. It is clear that there is a distinct separation of the amino acid profiles for the seminal plasmas of GF and PF bulls. According to our findings, phenylalanine could be considered as a freezability biomarker, and may be used as a cryoprotectant supplement. In addition, amino acid profiles of the seminal plasma could be used to determine the freezability phenotypes. These findings help us better understand the exact mechanisms of cryopreservation for sperm cells as well as other cell types.

## Data Availability Statement

The raw data generated from this study will be available through the corresponding author.

## Author Contributions

MU, AK, TD, and EM conceptualized the study. MU, TD, and MH curated the data. MU, TD, MH, and EM carried out the investigations. AK, BD, and ET provided the essential samples and phenotypic data. MU, TD, MH, AK, and EM wrote the original draft, and reviewed and edited the manuscript.

## Conflict of Interest

ET and BD employed by the company Alta Genetics, Inc. The remaining authors declare that the research was conducted in the absence of any commercial or financial relationships that could be construed as a potential conflict of interest.

## Footnotes

1 http://www.metaboanalyst.ca

2 https://cytoscape.org/
